# Real‐Time PCR–Based Detection of *Mycobacterium leprae* From Multiple Clinical Samples for Molecular Diagnosis of Leprosy in Côte D’Ivoire

**DOI:** 10.1155/jotm/9962633

**Published:** 2026-08-02

**Authors:** Bahou Roger Dehe, Aby Christiane Amon, Ouemela Venance Ban, Gogbé Jean-Luc Tiemoko, Henry Kouakou, Alain Dit Philippe Bidie, Mireille Dosso, Kakou Solange Ngazoa, N’Golo David Coulibaly

**Affiliations:** ^1^ UFR Marine Sciences, Laboratory of Oceanographic and Marine Resources, San Pedro University, San Pedro, Ivory Coast; ^2^ Molecular Biology Platform, Pasteur Institute of Côte D’Ivoire, BP 490 Abidjan, Abidjan, Ivory Coast, pasteur.ci; ^3^ UFR Agriculture, Fishery Resources and Agro-Industries, San Pedro University, San Pedro, Ivory Coast; ^4^ Follereau Raoul Institute of Côte D’Ivoire, Adzope, Ivory Coast; ^5^ UFR Biosciences, Laboratory of Biology and Health, Felix Houphouet-Boigny University, Abidjan, Ivory Coast

**Keywords:** côte d’Ivoire, leprosy, molecular diagnosis, *Mycobacterium leprae*, real-time PCR

## Abstract

**Background:**

Leprosy remains endemic in Côte d’Ivoire, with persistent Grade 2 disability cases indicating ongoing challenges in early diagnosis. Classical diagnosis relies on clinical examination and microscopy, which lacks sensitivity because *Mycobacterium leprae* cannot be cultured. In the context of the WHO “Zero Leprosy” strategy (2021–2030), molecular tools are needed to confirm cases, detect subclinical infections, and monitor transmission.

**Methods:**

We developed and evaluated a real‐time PCR protocol targeting the *M. leprae* 16S rRNA gene for molecular diagnosis. Clinical specimens (slit‐skin smears and nasal mucus) were collected from 20 clinically diagnosed leprosy patients in Côte d’Ivoire. DNA/RNA extraction was performed using both a semiautomated kit (NucliSENS) and a manual guanidinium thiocyanate (GuSCN) method. Real‐time PCR results were compared with direct microscopy (Ziehl–Neelsen staining). The choice of GuSCN was justified by its chaotropic properties, which inactivate mycobacteria, stabilize RNA, and improve biosafety.

**Results:**

Among 48 samples, microscopy detected only nine (18.8%) positive cases, all from multibacillary (MB) patients. In contrast, real‐time PCR detected 35 (72.9%) positives using the NucliSENS kit and 31 (64.6%) using the GuSCN method. In paucibacillary (PB) patients, PCR positivity reached 59.5%, whereas microscopy was nearly negative. Ear lobe exudates provided the best diagnostic yield.

**Conclusion:**

Real‐time PCR targeting 16S rRNA significantly improves *M. leprae* detection compared to microscopy, especially in PB forms. The manual GuSCN method is a reliable, low‐cost alternative for endemic settings. This approach can facilitate early case confirmation, subclinical infection detection, and support leprosy control efforts in Côte d’Ivoire within the WHO Zero Leprosy framework.

## 1. Introduction

Leprosy, a persistent infectious disease caused by *Mycobacterium leprae*, remains a public health challenge in many endemic countries, including Côte d’Ivoire. Although multidrug therapy (MDT) has substantially reduced the global burden, delayed diagnosis continues to lead to irreversible peripheral neuropathy and Grade 2 disabilities [1, 2]. In Côte d’Ivoire, despite a decline in reported cases from 1.169 in 2013 to 891 in 2015 [3], the proportion of patients presenting with Grade 2 disabilities increased in 2015 compared to 2011, indicating persistent delays in early detection [4]. According to the latest World Health Organization (WHO) reports, transmission continues, particularly in rural and underserved areas, and new case detection rates remain a concern. In response, the WHO launched the “Zero Leprosy” strategy (2021–2030), which emphasizes early diagnosis, interruption of transmission, and prevention of disability through integrated surveillance and molecular tools.

Conventional diagnosis in Côte d’Ivoire relies primarily on clinical examination and direct microscopy of slit‐skin smears using Ziehl–Neelsen staining. However, microscopy lacks sensitivity, especially in paucibacillary (PB) patients with low bacterial loads, and *M. leprae* cannot be cultured on axenic media [5]. Molecular diagnostic techniques, particularly real‐time PCR targeting species‐specific genetic markers such as the *16S rRNA* gene or *RLEP* repeat, have demonstrated enhanced sensitivity and specificity for *M. leprae* identification [6, 7]. Recent advances include multiplex qPCR using GMP‐grade reagents [8], loop‐mediated isothermal amplification (LAMP) for rapid field testing [9], and molecular viability assays that quantify bacterial transcripts (e.g., *esxA*, *hsp18*) to assess metabolic activity and monitor the treatment response [10]. Beyond simple detection, real‐time PCR targeting RNA (RT‐qPCR) can serve as an indirect indicator of bacterial viability, distinguishing live from dead bacilli, which is crucial for evaluating transmission risk and therapeutic efficacy. Furthermore, the choice of nucleic acid preservation method is critical: guanidinium thiocyanate (GuSCN)–based solutions inactivate mycobacteria, stabilize RNA, and improve biosafety during transport and handling, whereas ethanol‐based stabilizers may not guarantee complete inactivation [11].

In Côte d’Ivoire, the integration of molecular diagnostics into the leprosy control program remains limited. Few studies have evaluated low‐cost, manual extraction protocols suitable for resource‐limited settings in West Africa. Therefore, this study was designed to assess a real‐time PCR protocol targeting the *M. leprae 16S rRNA* gene using clinical specimens (slit‐skin smears and nasal mucus) from patients with suspected leprosy. The specific objectives were to (i) extract *M. leprae* DNA/RNA from different types of clinical samples using two extraction methods (semiautomated kit vs. manual GuSCN) and (ii) evaluate the performance of real‐time PCR for *M. leprae* detection compared to conventional microscopy, with the ultimate goal of providing a reliable, feasible molecular tool to support the WHO Zero Leprosy strategy in Côte d’Ivoire.

## 2. Materials and Methods

### 2.1. Study Setting

This study was conducted in Côte d’Ivoire at two institutions. Molecular analyses were performed at the Molecular Biology Platform (PfBM) of the Department of Techniques and Technology, Institut Pasteur of Côte d’Ivoire (IPCI, Adiopodoumé). Clinical diagnosis, microscopy, and sample collection were carried out at the Institut Raoul Follereau of Côte d’Ivoire (IRFCI, Adzopé).

### 2.2. Study Population and Sample Collection

The study population consisted of 20 patients clinically diagnosed with leprosy at IRFCI. Different types of clinical specimens were collected from each patient, including slit‐skin smears (from right and left ear lobes for Ziehl–Neelsen staining and microscopy) and nasal mucus. Each specimen was treated as an independent laboratory sample, resulting in a total of 48 samples analyzed. Conventional bacteriological diagnosis was performed by experienced biologists at IRFCI by direct smear microscopy.

### 2.3. Ethical Consideration

This study was approved by the National Ethics Committee for Research of Côte d’Ivoire: “Comité National d’Éthique de la Recherche de Côte d’Ivoire (CNER)” under Approval No.: N/Réf: N°140/MSHP/CNER‐km, which also covered a parallel study on the detection of drug resistance mutations by PCR. All participants signed an informed consent form after reading the study information notice.

### 2.4. Microscopy and Bacterial Index (BI)

Slit‐skin smears were stained using the Ziehl–Neelsen method. Acid‐fast bacilli (AFB) were examined under a light microscope at 1000 × magnification (oil immersion). At least 100 fields were examined per slide. The BI was calculated according to Ridley’s logarithmic scale (0–6+), where

• 1+ = 1–10 bacilli per 100 fields
• 2+ = 1–10 bacilli per 10 fields
• 3+ = 1–10 bacilli per field
• 4+ = 10–100 bacilli per field
• 5+ = 100–1000 bacilli per field
• 6+ = > 1000 bacilli per field

The histological index was recorded for each patient. Among the 9 microscopy‐positive samples, BI values ranged from 2+ to 5+ (mean BI in multibacillary (MB) patients: 3.2 ± 1.1). All PB patients had a BI of 0.

### 2.5. Nucleic Acid Extraction: Comparison of Two Methods

Two complementary methods were employed for nucleic acid extraction from clinical samples. A semiautomated approach using the NucliSENS extraction kit (bioMérieux, France) served as the reference method, and a manual method based on GuSCN was evaluated against it. Both procedures allowed the extraction of genomic DNA and total RNA for downstream molecular analysis.

### 2.6. Semiautomated Extraction (NucliSENS)

This method relies on the Boom et al. [12] technology, in which nucleic acids bind to magnetic silica particles under high‐salt conditions. Contaminants are removed through multiple washing steps using the NucliSENS miniMAG automated magnetic separator, and nucleic acids are eluted for PCR analysis. All steps were performed according to the manufacturer’s instructions.

### 2.7. Manual GuSCN Extraction

The GuSCN method was adapted from Yoshikawa et al. [11]. Choice of GuSCN: This chaotropic agent was selected because it inactivates RNases, stabilizes the transcriptional state, and effectively inactivates mycobacteria (including *M. tuberculosis* and *M. leprae*), thereby improving biosafety during transport and handling. Unlike ethanol‐based stabilizers, GuSCN ensures complete pathogen inactivation, which is particularly critical when processing samples from endemic regions with limited Biosafety Level 3 facilities. However, GuSCN may coprecipitate PCR inhibitors, and its performance can vary compared to commercial kits.

For the manual GuSCN extraction method, 200 μL of each clinical sample, previously pretreated with phosphate‐buffered saline, was mixed with 800 μL of lysis buffer containing 5 M GuSCN, 50 mM Tris‐HCl pH 8.0, 10 mM EDTA, 5% 2‐mercaptoethanol, and 2% Triton X‐100. The mixture was vortexed for 30 s and then incubated at room temperature for 20 min to ensure complete cell lysis and nucleic acid release. After incubation, the lysate was centrifuged at 10,000 × g for 3 min at 4°C, and the resulting supernatant was carefully transferred into sterile microtubes. This supernatant was mixed with 400 μL of isopropanol and centrifuged again at 10,000 × g for 5 min at 4°C to precipitate the nucleic acids. The pellet obtained was resuspended in 300 μL of elution buffer composed of 10 mM Tris‐HCl pH 8.0 and 1 mM EDTA. Subsequently, 110 μL of 3 M sodium acetate and 800 μL of absolute ethanol were added to the resuspended pellet, and the mixture was incubated at 4°C for 30 min to enhance nucleic acid precipitation. A final centrifugation step was performed at 10,000 × g for 5 min at 4°C. The resulting pellet was air‐dried at 50°C for 30 min, then resuspended in 150 μL of sterile distilled water, incubated at 55°C for 5 min to fully dissolve the nucleic acids, cooled to room temperature, and finally stored at −20°C until further PCR analysis.

### 2.8. DNA Quality Assessment

The quality and quantity of the extracted DNA were assessed using a Nanodrop spectrophotometer (Mettler Toledo UV5Nano) and by electrophoresis on 0.8% agarose gel. Ratios A260/A280 between 1.8 and 2.0 and A260/A230 > 2.0 were considered acceptable. Gel profiles showed high‐molecular‐weight bands consistent with genomic DNA, suitable for downstream molecular analysis.

### 2.9. Reverse Transcription quantitative PCR (RT‐qPCR) Targeting the *16S rRNA* Gene

Genomic DNA extracted from each method was used as the template for real‐time PCR targeting the 16S rRNA gene of *M. leprae*, a highly conserved and species‐specific region [13]. Rationale for targeting RNA: Although DNA detection confirms the presence of the bacterium, RNA quantification (via reverse transcription) serves as an indirect indicator of bacterial viability or metabolic activity. The *16S rRNA* is present at approximately 4000 copies per cell, enhancing sensitivity, and its detection reflects actively metabolizing bacteria, unlike genomic DNA, which may persist after cell death.

### 2.10. Primers and Probe

The primers and probe targeted a conserved fragment of approximately 171 bp. The reverse primer (ML16S rRNA‐R) is the antisense sequence. Sequences are presented in Table 1.

**TABLE 1:** Primers and probe sequences for the real‐time PCR detection of *M. leprae*.

Target	Primer/probe	Sequence (5′ ⟶ 3′)	Reference
16S rRNA	ML16S rRNA‐F (sense)	GCA TGT CTT GTG GTG GAA AGC	[14]
16S rRNA	ML16S rRNA‐R (antisense)	CAC CCC ACC AAC AAG CTG AT	[14]
16S rRNA	ML16S rRNA probe	FAM‐CAT CCT GCA CCG CA‐TAMRA	[14]

### 2.11. Reaction Conditions

Amplification reactions were performed in a final volume of 25 μL using an ABI 7300 Real‐Time PCR System (Applied Biosystems, USA). Each reaction mixture contained 5 μL of DNA/RNA template, 12.5 μL of 2 × PCR Master Mix, 4.8 μL of nuclease‐free water, 0.50 μL of ROX dye (Invitrogen), 0.50 μL of the 16S rRNA probe at a final concentration of 0.5 μM, 0.50 μL of each primer at a concentration of 10 μM each (resulting in a final concentration of 0.2 μM per primer), 0.20 μL of RNasin (Promega, USA), and 0.50 μL of SuperScript III reverse transcriptase enzyme (Invitrogen). The thermal cycling protocol began with reverse transcription for cDNA synthesis at 50°C for 2 min, followed by reverse transcriptase inactivation and initial denaturation at 95°C for 10 min. Subsequently, 40 amplification cycles were performed, each consisting of denaturation at 95°C for 15 s and annealing/extension at 60°C for 1 min.

Amplification curves were monitored in real time. Samples were considered positive when a specific amplification signal was observed within the threshold cycle (Ct) range.

### 2.12. Standard Curve

A standard curve was generated using serial 10‐fold dilutions (from 10^6^ to 10^1^ copies/μL) of a synthetic plasmid containing the *M. leprae* 16S rRNA target sequence. The amplification efficiency (*E*) was calculated as

(1) E=101∧−1/slope−×100.

The coefficient of determination (*R* ^2^) was > 0.99 and efficiency ranged from 90% to 110%. The limit of detection (LoD) was defined as the lowest concentration yielding positive amplification in ≥ 95% of replicates (determined as 10 copies/μL). All samples were run in duplicate; a Ct value < 38 was considered positive.

### 2.13. Data Analysis

Positivity rates were compared between microscopy and real‐time PCR using descriptive statistics. Sensitivity was calculated as (number of PCR‐positive samples/total samples) × 100. No inferential statistical tests were applied due to the small sample size.

## 3. Results and Discussion

### 3.1. Results

#### 3.1.1. Sociodemographic Characteristics

A total of 20 patients were included in the study, yielding 48 clinical samples. Among these, 11 were male (55%) and 9 were female (45%), giving a sex ratio of 1.22 (M/F). The mean age of participants was 32.7 ± 16.66 years ranging from 9 to 72 years. As observed in other endemic regions, males were more frequently affected than females. Table 2 presents the distribution of patients by age, sex, clinical form, and sample type.

**TABLE 2:** Distribution of patients by age, sex, clinical form, and sample type.

Patient id	Age (yrs)	Sex	Clinical form	Sample type^1^
M8	22	M	MB	001, 002, 003
M9	37	M	PB	002, 003
M10	09	M	PB	001, 003
M11	41	M	MB	001, 002
M12	49	M	PB	002, 003
M13	41	M	PB	002
M14	52	F	MB	001, 002, 003
M15	32	M	PB	001, 002
M16	10	F	PB	001, 002, 003
M17	23	M	PB	002
M18	24	F	PB	001, 002, 003
M19	23	F	PB	001, 002, 003
M20	45	F	MB	001, 002, 003
M21	55	M	PB	001, 002, 003
M22	38	F	PB	001, 002, 003
M23	09	M	PB	001, 002, 003
M24	23	F	PB	001, 002
M25	22	F	PB	001, 002, 003
M26	27	M	PB	001, 002
M27	72	F	PB	001, 002

*Note:* 002 = left ear lobe, 003 = nasal mucus. PB = paucibacillary, MB = multibacillary.

Abbreviations: F = female, M = male.

^1^Sample type codes: 001 = right ear lobe.

#### 3.1.2. Clinical Forms

Of the 20 patients, 16 (80%) had the PB form and 4 (20%) had the MB form, indicating a predominance of PB leprosy in the study population.

#### 3.1.3. Microscopy and BI

Direct microscopy after Ziehl–Neelsen staining revealed 9 positive samples (18.75%) out of 48, while 39 samples (81.25%) were negative. All microscopy‐positive samples originated exclusively from MB patients. Among these 9 positive samples, the BI according to Ridley’s logarithmic scale was distributed as follows: BI 2+ (1–10 bacilli per 10 fields) in 2 samples, BI 3+ (1‐10 bacilli per field) in 4 samples, BI 4+ (10‐100 bacilli per field) in 2 samples, and BI 5+ (100‐1000 bacilli per field) in 1 sample. The mean BI among MB patients was 3.2 ± 1.1. All PB patients had a BI of 0, confirming that their bacterial loads were below the microscopic detection threshold. Table 3 presents the microscopy results according to sample type.

**TABLE 3:** Microscopy results according to the sample type.

Sample type	Positive	Negative	Total
Ear lobe	8	27	35
Nasal secretion	1	12	13
Total	9	39	48

#### 3.1.4. Molecular Diagnosis (real‐time PCR)

Two DNA extraction methods were evaluated for real‐time PCR targeting the 16S rRNA gene of *M. leprae*: the semiautomated NucliSENS kit (bioMérieux) and the manual GuSCN method. Using the NucliSENS kit, 35 out of 48 samples (72.91%) tested positive. Using the GuSCN method, 31 out of 48 samples (64.58%) tested positive. Both methods performed satisfactorily, confirming that the GuSCN protocol is a reliable alternative for *M. leprae* nucleic acid extraction, particularly in resource‐limited settings. Table 4 summarizes the molecular diagnosis results by the extraction method.

**TABLE 4:** Molecular diagnosis by the extraction method (real‐time PCR).

Extraction method	Positive	Negative	Total
NucliSENS kit (bioMérieux)	**35**	**13**	**48**
Guanidinium thiocyanate (GuSCN)	**31**	**17**	**48**

*Note:* The bold values in Table 4 correspond to the number of samples tested for each experimental condition.

#### 3.1.5. PCR Results According to Clinical Form

When PCR results were analyzed according to clinical form using the NucliSENS reference extraction method, positivity reached 81.81% (9 out of 11 samples) in MB patients and 59.45% (22 out of 37 samples) in PB patients. Using the GuSCN method, positivity was 72.73% (8 out of 11 samples) in MB patients and 54.05% (20 out of 37 samples) in PB patients. These results demonstrate that real‐time PCR significantly increases diagnostic yield compared to microscopy, especially in PB patients where conventional microscopy is nearly always negative. Table 5 presents the molecular diagnosis results according to clinical form.

**TABLE 5:** Molecular diagnosis according to clinical form (real‐time PCR).

Clinical form	Number of samples	Positive	Negative
Multibacillary (mb)	11	9	2
Paucibacillary (PB)	37	22	15
Total	**48**	**31**	**17**

*Note:* The bold values in Table 5 correspond to the number of samples tested for each experimental condition.

#### 3.1.6. Standard Curve and Analytical Performance

A standard curve was generated using serial 10‐fold dilutions (from 10^6^ to 10^1^ copies/μL) of a synthetic plasmid containing the *M. leprae* 16S rRNA target sequence. The amplification efficiency was calculated as 94% (within the acceptable range of 90%–110%), the coefficient of determination (*R* ^2^) was 0.993, and the LoD was determined as 10 copies per reaction (positive amplification in ≥ 95% of replicates). These parameters confirm that the real‐time PCR assay is reliable, sensitive, and suitable for routine molecular diagnosis of leprosy.

## 4. Discussion

In this study, we evaluated a real‐time PCR (RT‐qPCR) protocol targeting the *16S rRNA* gene of *M. leprae* for the molecular diagnosis of leprosy in Côte d’Ivoire. Our results demonstrate that RT‐qPCR significantly improves detection compared to conventional microscopy, particularly in PB patients, and that a manual GuSCN extraction method is a reliable, low‐cost alternative to commercial kits. These findings have important implications for leprosy control in resource‐limited endemic settings within the framework of the WHO “Zero Leprosy” strategy (2021–2030).

Microscopy detected only 18.8% (9/48) of positive samples, all from MB patients, whereas RT‐qPCR detected 72.9% (35/48) using the reference NucliSENS extraction method and 64.6% (31/48) using the manual GuSCN method. This substantial difference is explained by the lower LoD of microscopy, which requires approximately 100 bacilli per field [15], whereas PCR can detect as few as 10 copies of target DNA per reaction (as determined by our standard curve, LoD = 10 copies/μL). In PB patients, who typically have bacterial loads below the microscopic threshold [16], PCR positivity reached 59.5% (22/37) compared to nearly 0% by microscopy. These results are consistent with Wen et al. [17], who reported 36.4% PCR positivity in PB patients, and with Sarath et al. [7], who demonstrated that qPCR targeting *RLEP* had higher sensitivity than conventional PCR for tissue specimens.

We targeted the *16S rRNA* gene rather than genomic DNA for two complementary reasons. First, each *M. leprae* cell contains approximately 4000 copies of *16S rRNA* [18], providing enhanced analytical sensitivity compared to single‐copy DNA targets. Second, RNA detection via reverse transcription (RT‐qPCR) serves as an indirect indicator of bacterial viability or metabolic activity. Unlike DNA, which can persist after cell death, RNA is rapidly degraded in nonviable bacteria, so the presence of specific RNA transcripts reflects actively metabolizing organisms. This distinction is critical for monitoring treatment response and transmission risk, as only viable bacteria contribute to disease propagation and drug resistance selection. However, we acknowledge that *16S rRNA* is relatively stable compared to messenger RNA (e.g., *esxA*, *hsp18*), and its detection may overestimate viability in some contexts. Future studies should compare *16S rRNA*‐based viability assessment with shorter‐half‐life mRNA targets to refine molecular monitoring of therapeutic efficacy [10].

The manual GuSCN method performed satisfactorily, with 64.6% positivity compared to 72.9% for the commercial NucliSENS kit. This difference is acceptable for resource‐limited settings where the cost of commercial kits may be prohibitive [19]. More importantly, we chose GuSCN not only for its nucleic acid extraction efficiency but also for its chaotropic properties, which inactivate mycobacteria and RNases, stabilize the transcriptional state, and improve biosafety. GuSCN‐based buffers (e.g., GTC‐TCEP) have been shown to effectively inactivate *M. tuberculosis*, whereas some ethanol‐based stabilizers do not guarantee complete inactivation [11]. For *M. leprae*, which cannot be cultured, chemical inactivation is essential when processing clinical samples outside Biosafety Level 3 facilities. Nevertheless, GuSCN may coprecipitate PCR inhibitors, and its performance can vary depending on sample type and storage conditions. Laboratories adopting this method should include internal amplification controls and validate inhibition rates locally.

Our findings align with recent advances in leprosy molecular diagnostics. Manta et al. [8] developed a GMP‐compliant multiplex qPCR assay suitable for routine diagnostic laboratories, demonstrating that qPCR can be standardized for clinical use. Joshi et al. [9] described a LAMP assay for rapid field testing; while LAMP offers faster turnaround and does not require thermal cyclers, it is less quantitative than real‐time PCR and may have lower specificity in low‐bacillary cases. Sarath et al. [7] confirmed that qPCR targeting RLEP is highly sensitive for tissue specimens, but the RLEP target is absent from some *M. leprae* strains, whereas 16S rRNA is universally present. Our approach using 16S rRNA thus offers a balance between sensitivity, specificity, and universal detection. Lopes‐Luz et al. [10] have recently highlighted that serological tests remain insufficient for early diagnosis and emphasized that molecular methods, particularly those assessing viability, are essential for monitoring transmission and treatment response.

Ear lobe exudates provided the highest molecular diagnostic yield, with 88.9% of microscopy‐positive samples originating from ear lobes. In contrast, nasal mucus showed low positivity (11.1% of microscopy‐positive samples). This observation is consistent with a Brazilian study [20] reporting only 1.7% *M. leprae* positivity in nasal swabs. Nasal shedding of *M. leprae* is generally transient and more pronounced in untreated lepromatous patients, making nasal samples less reliable for routine diagnosis. Therefore, we recommend ear lobe slit‐skin smears as the primary sample type for molecular diagnosis in this setting.

The WHO “Zero Leprosy” strategy (2021–2030) has three pillars: early diagnosis and prompt treatment, interruption of transmission, and prevention of disability. Our RT‐qPCR protocol directly supports all three pillars. First, by detecting PB cases that are missed by microscopy, PCR enables earlier diagnosis and treatment initiation, preventing irreversible nerve damage and Grade 2 disabilities. Second, the ability to assess bacterial viability via RNA detection helps identify active transmission foci and monitor the effectiveness of postexposure prophylaxis. Third, the manual GuSCN method is feasible in district laboratories with basic equipment, facilitating decentralized molecular surveillance. In Côte d’Ivoire, where the proportion of Grade 2 disabilities increased between 2011 and 2015 [4], integrating molecular diagnostics into the national leprosy control program could reverse this trend and accelerate progress toward zero leprosy.

Several limitations should be acknowledged. First, the sample size was small (20 patients, 48 specimens), and the study was conducted at a single reference center, which may limit generalizability. Second, we did not perform sequencing to confirm amplicon specificity or to identify *M. leprae* genotypes. Third, although we used RT‐qPCR to detect 16S rRNA as a viability indicator, we did not directly compare RNA signals with DNA signals or with BI to quantify the relationship between transcriptional activity and bacterial load. Fourth, we did not include a panel of negative controls from healthy endemic controls or patients with other skin diseases, so specificity could not be formally calculated. Fifth, the GuSCN method showed slightly lower sensitivity than the commercial kit, and inhibition rates were not systematically assessed. Future studies should address these limitations by including larger sample sizes, multicenter validation, direct comparison of RNA vs. DNA detection, and formal specificity testing.

To build on these findings, we propose the following perspectives. First, evaluate the RT‐qPCR protocol for detecting subclinical infections in household contacts of leprosy patients, as these individuals represent a key reservoir for continued transmission. Second, adapt the method for viability assessment using shorter‐half‐life mRNA targets such as *esxA* or *hsp18*, which may provide more accurate real‐time monitoring of treatment response. Third, sequence positive amplicons to identify *M. leprae* genotypes and monitor potential drug resistance mutations, particularly given the parallel study on drug resistance approved by the ethics committee. Fourth, conduct a cost‐effectiveness analysis comparing the GuSCN manual method with commercial kits to guide national policy decisions. Finally, transfer the validated protocol to regional laboratories in Côte d’Ivoire to support decentralized surveillance aligned with the WHO Zero Leprosy roadmap.

## 5. Conclusion

This study demonstrates that real‐time PCR targeting the 16S rRNA gene of *M. leprae* significantly improves diagnostic sensitivity compared to conventional microscopy in Côte d’Ivoire. Among 48 clinical specimens, microscopy detected only 18.8% of positive cases, all from MB patients, whereas real‐time PCR detected 72.9% using a commercial extraction kit and 64.6% using a manual GuSCN method. Notably, in PB patients, who typically have bacterial loads below the microscopic threshold, PCR positivity reached 59.5%, demonstrating its value for early diagnosis before irreversible nerve damage occurs. The manual GuSCN method proved to be a reliable, low‐cost alternative to commercial kits, with the additional advantage of inactivating mycobacteria and stabilizing RNA, thereby improving biosafety and enabling downstream viability assessment. Ear lobe slit‐skin smears provided the highest diagnostic yield, while nasal mucus was less reliable for routine testing. These findings directly support the WHO “Zero Leprosy” strategy (2021–2030) by enabling earlier case confirmation, facilitating the detection of subclinical infections, and providing a foundation for monitoring the treatment response and transmission interruption. For Côte d’Ivoire, where the proportion of Grade 2 disabilities increased between 2011 and 2015, integrating this molecular protocol into the national leprosy control program could reverse diagnostic delays and accelerate progress toward elimination. Future research should focus on validating the assay in household contacts, comparing RNA‐based viability indicators with shorter‐half‐life mRNA targets such as *esxA* or *hsp18*, sequencing positive amplicons to monitor genotypes and drug resistance, and conducting cost‐effectiveness studies to guide decentralized implementation. Ultimately, the adoption of real‐time PCR using the manual GuSCN method in resource‐limited endemic settings represents a feasible and impactful step toward zero leprosy.

## Funding

No funding was received for this research.

## Conflicts of Interest

The authors declare no conflicts of interest.

## Data Availability

The data that support the findings of this study are available from the corresponding author upon reasonable request.
